# 938. Azole Resistant *Aspergillus* Species in Lung Transplant Recipients: A 10 Year Experience

**DOI:** 10.1093/ofid/ofab466.1133

**Published:** 2021-12-04

**Authors:** Nicholas A Marschalk, Palash Samanta, Eun Jeong Kwak, Minh-Hong Nguyen

**Affiliations:** 1 The Ohio State University Medical Center, Columbus, Ohio; 2 University of Pittsburgh, Pittsburgh, PA

## Abstract

**Background:**

Invasive aspergillosis (IA) causes significant morbidity and mortality in lung transplant (LTx) recipients. Antifungal resistance in Aspergillus species is on the rise globally with specific concern in Europe related to the TR34/L98H mutation in the cyp51A enzyme that induces pan-azole resistance. Azole exposure is a known risk factor for development of resistant Aspergillus, but this is less well described in LTx population.

**Methods:**

We reviewed the electronic medical records of LTx patients with respiratory cultures positive for Aspergillus species known to be inherently resistant or any Aspergillus species tested to be resistant to one or more triazole from 2010 to 2019. For available isolates, Sanger sequencing was performed on cyp51A with primers targeting the promoter region and 3 known hotspot areas.

**Results:**

Twenty eight patients met inclusion criteria and 2.7% (28/1026) Aspergillus isolates were azole-resistant during study period (Figure 1). Median time from LTx to resistant Aspergillus growth was 196 days (range 14 - 3146). There was a cluster of positive cultures within 1-year post-Tx period (13/28). Azole exposure varied, from 7 to 2443 days (median 128). There was no change in incidence over the study period. The most common species was Aspergillus calidoustus (Figure 2). Twenty cases were deemed colonization, vs 5 probable IFI and 3 proven IFI. Mortality of IFI with resistant Aspergillus was 38% (3/8), higher than azole-susceptible IA (p=0.05). Twelve isolates were available for sequencing; none carried TR34/L98H mutation. There was wide variation in mutations, ranging from 1 to 12 point mutations in the cyp51 enzyme, many of them SNPs previously described as engendering an azole resistant phenotype (Figure 3).

Aspergillosis-free Survival by Resistant and Susceptible Isolates

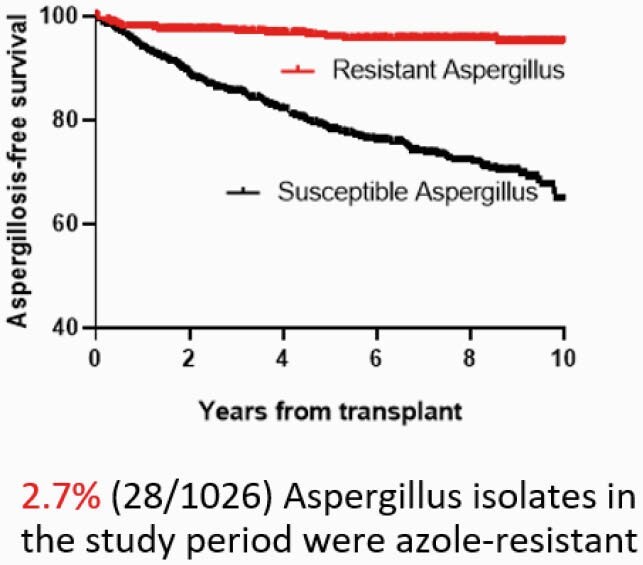

Development of azole-resistant aspergillus colonization/infection was rare relative to common occurrence of colonization/infection with susceptible isolates in the lung transplant recipient population

Azole Resistant Aspergillus Species Isolated from Lung Transplant Recipients

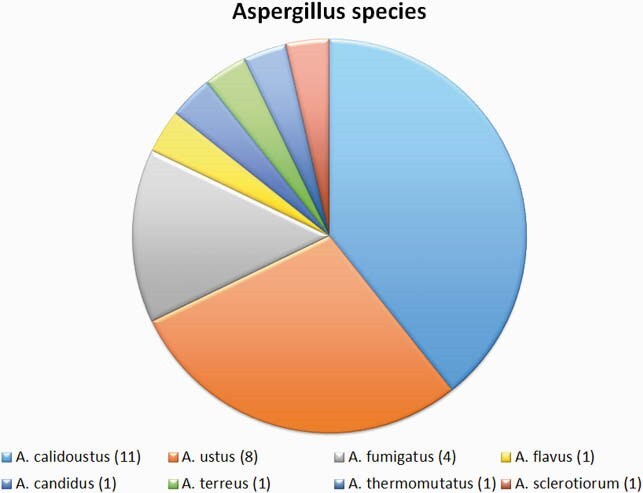

The most commonly isolated species were A. calidoustus and ustus. Only 4/28 isolates were A. fumigatus.

Azole Resistant Aspergillus Genotypic Analysis

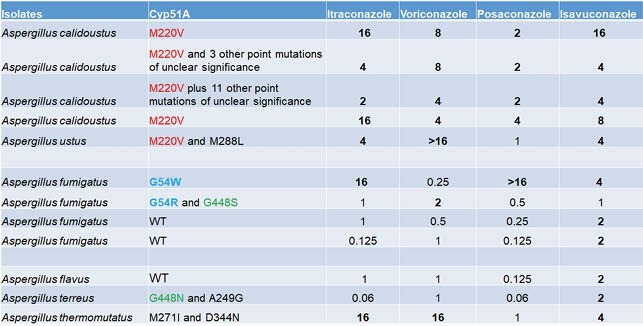

Sequenced isolates showed wide variability in point mutations. M220V, G54, and G448 were the only mutations observed more than once. 3 isolates were wild type.

**Conclusion:**

Azole resistant Aspergillus infections remain an uncommon problem in LTx. The majority of isolates were deemed colonization, but mortality was high when IFI was present. Most isolates had mutations within the hot spot regions of cyp51A known to induce azole resistance. There were no TR34/L98H mutants found in our patient population.

**Disclosures:**

**Minh-Hong Nguyen, MD**, **Merck** (Grant/Research Support)

